# QTL-seq analysis of seed protein quantity and quality traits in two soybean recombinant inbred line populations

**DOI:** 10.3389/fpls.2026.1771028

**Published:** 2026-04-15

**Authors:** Jianyu Zhang, Sepideh Torabi, Milad Eskandari

**Affiliations:** 1 Department of Plant Agriculture, University of Guelph, Guelph, ON, Canada; 2 PreFer Industries Ltd, Burlington, ON, Canada; 3 Graduate Programs in Bioinformatics, University of Guelph, Guelph, ON, Canada

**Keywords:** bulked segregant analysis, protein quality, QTL-seq, seed amino acids, seed protein, soybean

## Abstract

**Introduction:**

Soybean [*Glycine max* (L.) Merr.] is an important source of plant-based dietary protein, yet its nutritional quality is frequently constrained by deficiencies in the concentrations of amino acids cysteine (Cys), methionine (Met), threonine (Thr), and lysine (Lys). The identification of quantitative trait loci (QTL) associated with seed protein quantity and quality traits are essential for assisting breeders in the development of improved cultivars. This study aimed to identify and validate QTL associated with these five seed traits using novel genetic backgrounds.

**Methods:**

Two recombinant inbred line (RIL) populations, POP179 and POP180, were derived from crosses between unique high-protein fast neutron-induced mutants. Phenotypic evaluations were conducted across four site-year environments in southern Ontario, Canada. A modified QTL-sequencing (QTL-seq) method was implemented to detect genomic regions with unequal parental contributions that likely harbour putative QTL.

**Results:**

All five seed traits exhibited high broad-sense heritability (0.81 to 0.90) and significant positive correlations. Three stable putative QTL were identified and validated on chromosomes 1 (27.1–32.3 Mbp), 2 (8.6–10.8 Mbp), and 15 (16.7–20.8 Mbp) in both populations.

**Discussion:**

The co-localization of genomic regions associated with multiple traits suggests a shared genetic architecture governing seed composition. Identified candidate genes, such as glutamine synthase and peptide transporters, are likely involved in nitrogen assimilation and nutrient loading during seed development. These findings provide additional resources for marker-assisted selection and genomic prediction that can support the development of soybean cultivars with superior nutritional profiles.

## 1 Introduction

Soybean [*Glycine max* (L.) Merr.] is an important source of protein for human and livestock diets (Qin et al., 2022; Pope et al., 2023; Cordoba et al., 2025). A market trend analysis released by Agriculture and Agri-Foods Canada (Agriculture and Agri-Foods Canada, 2024) in 2023 found that the Canadian market for non-animal derived proteins reached 43.6 thousand tons (Haidar et al., 2025). Soy protein concentrates and isolates together account for over 60% of the non-animal derived proteins market share (Agriculture and Agri-Foods Canada, 2024). Soybean meal serves as a major source of protein for livestock feed. Over 281 million tons of soybean meal were produced globally in the 2024/2025 marketing year (USDA Foreign Agricultural Service, 2025). In the United States, dairy, swine, and poultry production consumed over 90% of soybean meal, according to the United Soybean Board (2023). Pope et al. (2023) estimated that a 1% increase in soybean meal crude protein concentration would increase the value of soybean meal by USD$10.27 and $12.62 per ton for swine and poultry feed, respectively. Protein accounts for 30 to 40% of the dry soybean seed weight on average. Compared to egg white protein, soybean seed protein is deficient in the amino acids cysteine (Cys), methionine (Met), threonine (Thr), and lysine (Lys) (George and de Lumen 1991); among which Met, Thr, and Lys are essential for healthy human and livestock diets.

Investigating the genetic control of seed protein and amino acid concentrations may identify quantitative trait loci (QTL) potentially useful for breeding cultivars with enhanced nutritional and economic values. Seed protein is an agronomically-important trait for soybean, and its genetic basis has been the subject of extensive investigations and have yielded QTL across most chromosomes (Grant et al., 2010; Diers et al., 1992; Fasoula et al., 2004; Nichols et al., 2006; Pathan et al., 2013). Despite their contributions to seed protein quality and nutritional value, the concentrations of individual amino acids, including Cys, Met, Thr, and Lys, have received notably less effort in comparison. Genomic regions that are associated to both Cys and Met have been reported by Panthee et al. (2006); Ramamurthy et al. (2014), and Malle et al. (2020). These QTL suggest that the seed concentrations of the two sulfur-containing amino acids are partially linked. Recent QTL-seq and GWAS studies have identified novel loci and candidate genes for sulfur-containing amino acids, such as those involving cysteine β-lyase homologs and methyltransferases, further supporting their genetic co-regulation and potential for marker-assisted selection to improve nutritional profiles (Zhong et al., 2022; Van et al., 2025; Taliercio et al., 2024). These findings, alongside updated genomic resources from wild soybean accessions, underscore the promise of integrating advanced mapping with breeding to enhance amino acid-specific QTL discovery and soybean seed quality.

Seed protein QTL *cqProt-003* on chromosome 20, derived from PI 468916, was among the first major loci mapped in soybean (Diers et al., 1992). A protein QTL from the Korean cultivar Danbaekkong (Benning × Danbaekkong population) also mapped to the same region (Warrington et al., 2015), where the favourable Danbaekkong allele explained up to ~55% of the phenotypic variation for seed protein. Subsequent linkage and GWAS work (Teng et al., 2017) reported multiple loci, including *qPR-3* (chr12, Sat_122), *qPR-5* (chr17, Satt543), and *qPR-8* (chr20, Satt614) in a RIL population of 129 F_5:8_ lines exhibiting significant additive effects on protein concentration. A major protein QTL on chr2 (GBS02_47440443K) was reported by Clevinger et al. (2023) that explained up to 56.8% of variations in seed protein within a biparental population that was derived by crossing the high protein PI 399084 with the low protein PI 507429. Early work on amino-acid composition (Panthee et al., 2006) identified four QTL associated with Cys concentration across linkage groups D1a, F, and G (chr1, 13, and 18, respectively) and three QTL linked with Met concentration on linkage groups F, G, and M (chr14, 18, and 7, respectively). Additionally, three QTL on linkage groups D2, F, and M (chr17, 13, and 7, respectively) were associated with both Cys and Met concentrations (Panthee et al., 2006). Souza et al. (2023a) reported Cys and Met QTL on chromosomes 3, 6, 10, and 15. Cys and Met on chromosome 8 were found by Vaughn et al. (2014) and Singer et al. (2022) in separate genome-wide association studies on diverse germplasms. Warrington et al. (2015) reported four protein QTL (chr 14, 15,17, 20), one Cys QTL (chr10), four Met QTL (chr6, 9, 10, 20), four Thr QTL (chr1, 9, 17, 20), and two Lys QTL (chr8, 20). These findings highlight chromosome 20 is a hotspot for seed protein and amino acids QTL. Notably, a single Cys QTL on chromosome 10 overlapped with a Met QTL, both exhibiting major effects, suggesting that co-mapping these sulfur-containing amino acids may enhance detection efficiency (Warrington et al., 2015). However, Warrington et al. (2015) noted that their identified Cys and Met QTL did not share chromosomal regions with those reported by Panthee et al. (2006). Additional research into the genetic control of seed protein and amino acids concentrations may assist breeders in developing cultivars with improved nutritional value and would benefit the producers and consumers of soybean-derived food products.

Genetic linkage maps with high marker density and uniform marker coverage greatly enhance the success of QTL detection (Collard and Mackill, 2008). However, since the entire mapping population must be screened with markers for linkage calculations, constructing high-quality linkage maps can be both costly and time-consuming. QTL-sequencing (QTL-seq) is an alternative biparental mapping technique developed by Takagi et al. (2013) that combines bulked segregant analysis (BSA) with high-throughput genotyping. In brief, QTL-seq detects QTL in genomic regions with unequal parental contributions. Two parents with contrasting values of a trait are crossed to generate a segregating offspring population. Offspring individuals with extreme contrasting values for a trait are grouped into low and high trait value bulks. The genotypes of individuals in each bulk are aligned to a reference parent to identify genomic regions with unequal parental contributions that are likely to harbour major QTL associated with the contrasting trait. By sequencing only the two parents and sampled individuals from the two bulks, rather than the entire population, time and cost are significantly reduced compared to biparental linkage mapping (Takagi et al., 2013). QTL-seq has been applied to detect QTL for numerous traits across multiple species, such as chickpea seed weight (Singh et al., 2016), capsaicinoid biosynthesis in hot peppers (Park et al., 2019), and powdery mildew resistance in cucumbers (Zhang et al., 2021). QTL-seq has also been used to detect QTL in soybean (da Silva et al., 2019; Wang et al., 2021). However, to the best of the authors’ knowledge, there are currently no published reports on the application of QTL-seq for identifying QTL associated with soybean seed protein quality traits.

The primary objective of this study was to identify QTL associated with seed protein, Cys, Met, Thr, and Lys concentrations in soybean seeds. This was achieved using the QTL-seq approach in two recombinant inbred line (RIL) populations evaluated across four site-year environments in southern Ontario, Canada.

## 2 Materials and methods

### 2.1 Plant materials and phenotyping

Three high-protein, fast neutron-induced mutants, MAA26, MAA273, and MAA162, were developed by the University of Minnesota, each exhibiting distinct and elevated seed concentrations of Cys, Met, Thr, and Lys (**Supplementary Table 1**). These three mutants were used as parents to develop two F_4:7_ RIL populations to identify and validate QTL for seed protein, Cys, Met, Thr, and Lys concentrations. Population 179 (POP179) consisted of 153 RILs derived by crossing between MAA26 and MAA273, Population 180 (POP180) consisted of 139 F_4:7_ RILs derived by crossing MAA273 and MAA162. Summary statistics of seed protein and amino acids concentrations for POP179 and POP180 are provided in **Supplementary Table 2**.

The two RIL populations and parents were planted over the growing seasons of 2020 and 2021 in four location-year environments across southern Ontario: Chatham 2020, Palmyra 2020, Ridgetown 2020, and Ridgetown 2021. Each of the RILs were planted as a single 4.2-meter-long row with 100 seeds in a randomized complete block design with two replications.

Seeds from each RIL were harvested at full maturity. Seed composition traits, expressed as a percentage on a dry matter basis, were measured using near-infrared spectroscopy (NIRS) with the DA 7250 At-line NIR Instrument (PerkinElmer Canada, Winnipeg, MB) (PerkinElmer n.d.). Measurements were conducted on samples of 100 seeds per RIL, using calibrations provided by the manufacturer (PerkinElmer Canada, Winnipeg, MB).

### 2.2 Genotyping

Fresh leaf tissue samples were collected from each RIL and parent from the Ridgetown 2020 site and preserved by freeze-drying. Genomic DNA from the RIL populations and parents were extracted from the freeze-dried tissue samples using the NucleoSpin Plant II DNA Extraction Kit (Macherey-Nagel, Duren, Germany) according to manufacturer’s instructions (Macherey-Nagel n.d.). DNA sample quality and concentration was assessed using a NanoDrop 1000 Spectrophotometer and Qubit 2.0 Fluorometer (Thermo Fisher Scientific Waltham, MA, USA). DNA samples of all RILs and parents were sent to the Institute of Integrative Biology and Systems at Université Laval for genotyping-by-sequencing (GBS; Laval, Quebec, Canada) using the Illumina NovaSeq 6000 platform (Illumina Incorporated, San Diego, California). Variant calling was conducted through the Fast-GBS pipeline developed by Torkamaneh et al. (2017). Briefly, the raw sequencing data was first demultiplexed using Sabre, then trimmed and cleaned using Cutadapt (Najoshi, 2010; Martin, 2011). The cleaned sequences are aligned to the Gmax_275_v2 reference genome obtained from Phytozome (https://phytozome.jgi.doe.gov, Goodstein et al., 2012) for variant calling by Platypus; Rimmer et al., 2014; Torkamaneh et al., 2017). Variants called from the Fast-GBS pipeline were further filtered to remove SNPs with >10% missing data and heterozygosity, and minor allele frequency >0.05. In total, 66,554 SNPs were identified across 20 chromosomes, with the number of SNPs ranging between 1,566 to 7,409 per chromosome.

### 2.3 Statistical analysis

R version 4.0.3 (R Core Team, 2022), and RStudio version 1.4.1103 (RStudio Team, 2020) software were used to perform all statistical analyses. The multi-environment trial data of each population was subjected to analysis of variance (ANOVA). The data for seed protein concentration as well as amino acids Cys, Met, Thr, and Lys concentrations were analyzed using linear mixed models in two stages. The first stage assessed random effects of genotype and blocking in individual environments and calculated the genotype mean estimates in each environment as environmental best linear unbiased predictors (BLUPs). The second stage analyzes the random effect of genotype, environment, and genotype by environment interactions (GEI); genotype mean estimates across all environments are calculated as GEI BLUPs. The lme4 package (Bates et al., 2015) was used to fit linear mixed models; the lmerTest package (Kuznetsova et al., 2017) was used to test for significance of random effects; Environmental and GEI BLUPs were extracted with the lme4 package (Bates et al., 2015). Broad-sense heritability values of all five seed traits were estimated using the following equation:


$$H^{2}=\sigma_{G}^{2}/[\sigma_{G}^{2}+(\sigma_{GEI}^{2}/e)+\sigma_{Error}^{2}/re]$$

where $\;H^{2}$ is the estimated broad-sense heritability, $\sigma_{G}^{2}$ is the genotypic variance, $\sigma_{GEI}^{2}$ is the GEI variance, $\sigma_{Error}^{2}$ is the error variance, r is the number of replications, and e is the number of environments (Fehr, 1987).

Correlation matrices were constructed using the psych package (Revelle, 2022). Pearson’s correlation coefficients were calculated for all pairwise relations between the five seed traits based on the calculated GEI BLUPs to assess relationships.

### 2.4 QTL-seq

This study applied a modified QTL-seq analysis based on the original method developed by Takagi et al. (2013). Individual RILs within each of the two populations that show extreme contrasting phenotype values were selected for QTL-seq analysis. The RILs were selected separately for each of the five seed components: protein, Cys, Met, Thr, and Lys. The RILs were screened based on their measured seed component concentrations in each of the four test environments, as well as their environmental and GEI BLUPs. For every seed trait, 20 RILs with overall lowest measured trait values and BLUPs were selected to form the low bulk; likewise, the high bulks of each target trait are formed by selecting 20 RILs with overall highest measured trait values and BLUPs. Each of the five traits had its own set of low and high bulks; bulk selection was performed separately for POP179 and POP180. The RILs within each bulk are listed along with their combined BLUPs in **Supplementary Tables 3**, **4** for POP179 and POP180, respectively.

The SNP data of the low and high bulks were aligned against a reference parent to determine SNP allele frequency at each locus. The parent that displayed higher trait values was selected as the reference. For POP179, the low and high bulks were aligned against MAA26; bulks of POP180 were aligned against MAA162. The SNP allele frequency is calculated as the number of RILs within the bulk with a SNP allele that is polymorphic to the reference parental allele, divided by 20, the total number of RILs within each bulk.


$$SNP\;allele\;frequency=\;\frac{number\;of\;RILs\;with\;polymorphic\;allele}{20}$$

A locus with SNP allele frequency of 0 indicated that all RILs in the bulk share the same allele with the reference parent at that position; frequency of 1 indicated that all RILs share the same allele with the other parent; frequency of 0.5 indicated equal allele contribution from both parents. Loci with frequencies of 0.3 and lower were removed to reduce noise in subsequent calculations.

For each bulk, the SNP allele frequency at each locus was plotted against its corresponding chromosomal position to draw SNP allele frequency plots for every chromosome. These plots enabled the assessment of genome contribution from each parent to the offspring RILs. Comparing the SNP allele frequency plots of the low and high bulks can reveal regions with unequal parental contributions, and these regions may contain QTL associated with the contrasting phenotype between the two bulks. In the SNP allele frequency plots, genome regions with differing parental contributions would deviate from 0.5. To better visualize contrasts between the plots of low and high bulks, the SNP allele frequency of the low bulk is subtracted from that of the high bulk at every locus to create the Δ SNP allele frequency plot.


$$\Delta \;SNP\;allele\;frequency=\;SNP\;allele\;frequency_{high\;bulk}-\;SNP\;allele\;frequency_{low\;bulk}$$

In the Δ SNP allele frequency plots, regions with Δ frequencies near zero indicated similar allele frequencies between the two bulks; peaks and troughs indicate regions that are likely to contain QTL associated with the contrasting phenotypic trait between the two bulks. Peaks indicated regions where RILs in the high bulk have greater reference parental allele contributions compared to the low bulk; in contrast, troughs are regions where RILs in the low bulk contain more reference parental alleles compared to the high bulk.

**Figure 1:**
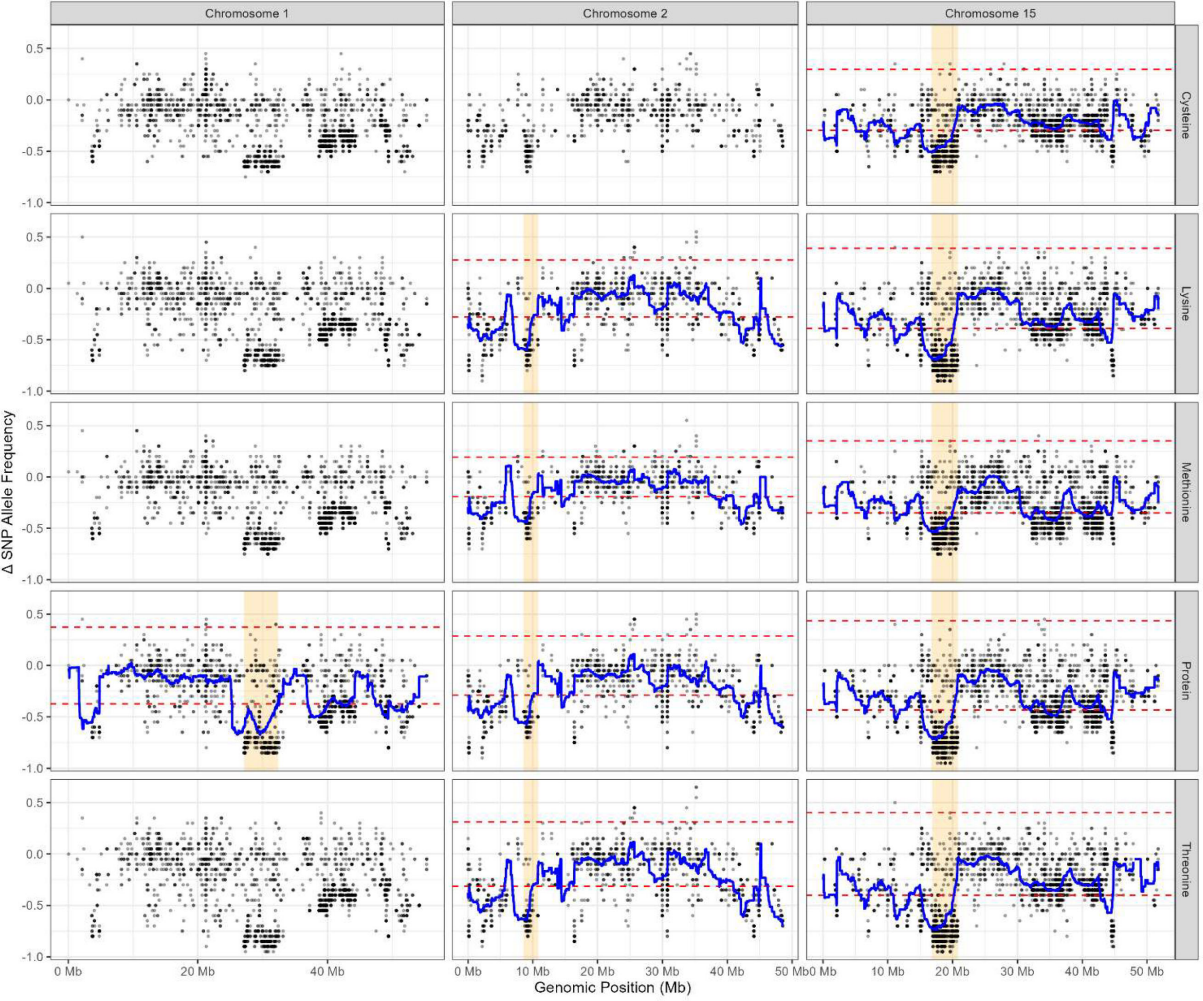
The Δ single nucleotide polymorphism (SNP) allele frequency plot of 20 soybean chromosomes based on modified QTL-seq analysis of POP179 for seed concentrations (% dry seed weight) of protein, cysteine, methionine, threonine, and lysine. The x-axis depicts the chromosomal position of the allele in mega-basepairs (Mb). The y-axis represents ΔSNP allele frequencies of each locus, obtained by subtracting the SNP allele frequency in the low bulk from that of the high bulk. Blue lines represent the results of sliding window analyses of each chromosome with a window width of 2Mbp and sliding length of 10Kbp. Red dashed lines represent the 99.9% confidence interval.

**Figure 2:**
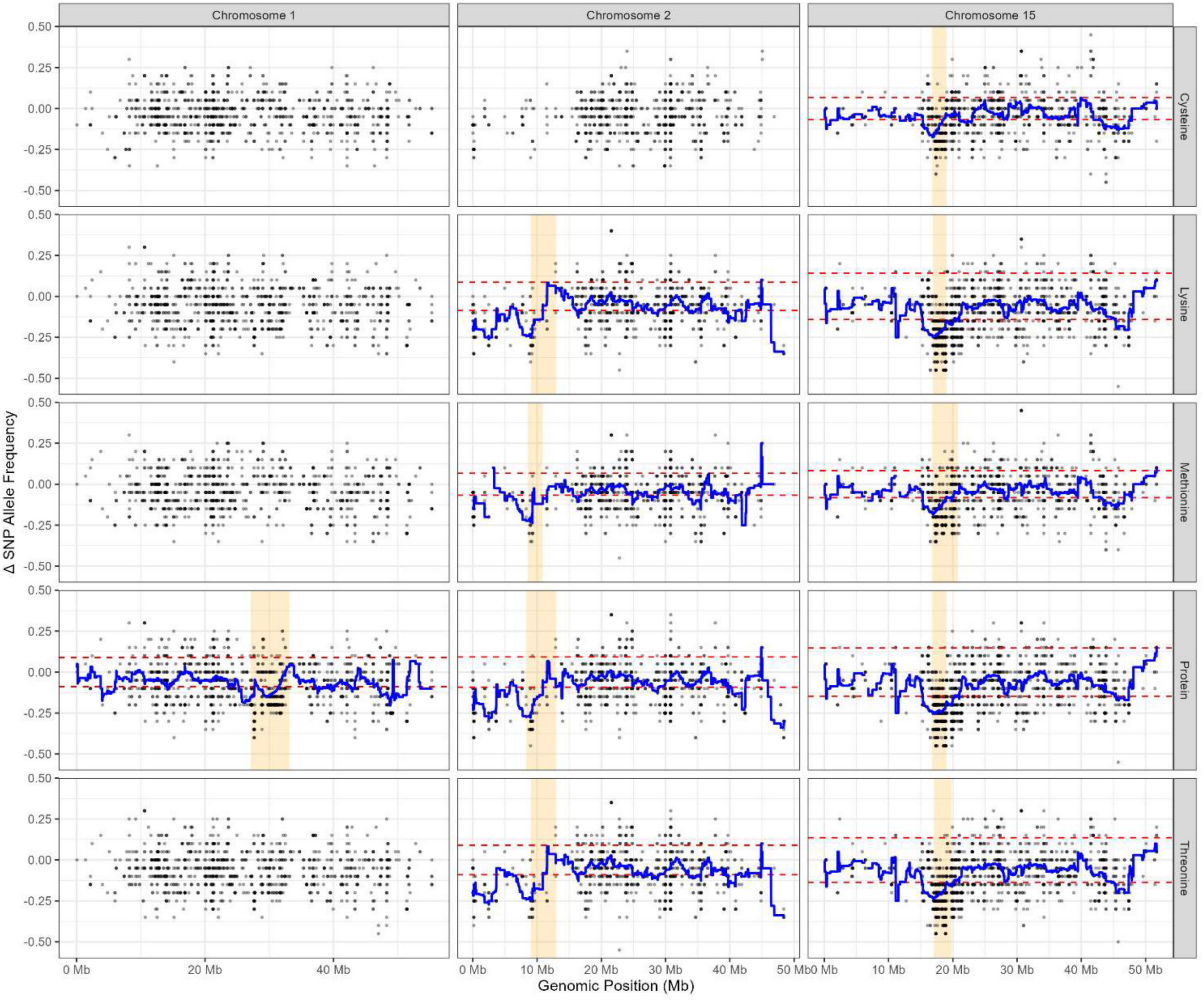
The Δ single nucleotide polymorphism (SNP) allele frequency plot of 20 soybean chromosomes based on modified QTL-seq analysis of POP180 for seed concentrations (% dry seed weight) of protein, cysteine, methionine, threonine, and lysine. The x-axis depicts the chromosomal position of the allele in mega-basepairs (Mb). The y-axis represents ΔSNP allele frequencies of each locus, obtained by subtracting the SNP allele frequency in the low bulk from that of the high bulk. Blue lines represent the results of sliding window analyses of each chromosome with a window width of 2Mbp and sliding length of 10Kbp. Red dashed lines represent the 99.9% confidence interval.

Sliding window analysis was applied to the Δ SNP allele frequency data to visualize trends and to assist identifying genomic regions potentially harbouring QTL. The window width was set to 2Mbp with sliding length of 10Kbp, adhered to the parameters of Takagi et al. (2013) and Zhang et al. (2021), such that a mean Δ allele frequency is calculated for all SNP loci within the first 1 - 2,000,000bp window, then subsequently for loci within the 10,001 - 2,010,001bp window, hereafter repeated to the end of the chromosome. Sliding window analyses were carried out for all 20 chromosomes and repeated for all five traits. The mean Δ SNP allele frequencies were drawn as lines onto the Δ SNP allele frequency plots (**Figure 1**; **Supplementary Figure 1** for POP 179; **Figure 2**; **Supplementary Figure 2** for POP180). Peaks and troughs in the mean Δ frequency line were considered as regions potentially harbouring QTL.

Confidence interval of each chromosome was calculated to determine statistical significance of potential regions.


$$CI=|\bar{x}|\pm t\times \frac{\sigma }{\sqrt{n}}$$

where:

$\left|\bar{x}\right|$ is the absolute value of the mean Δ SNP allele frequency of the entire chromosome,

*t* is the t-value at 99.9% confidence level,

*σ* is the standard deviation in Δ SNP allele frequency,

and *n* is the number of SNP loci in the chromosome.

The 99.9% CI defines the statistical threshold. To mitigate the detection of false positive regions, SNPs were further filtering based on consistent frequency. Genomic regions were only validated as QTL if they exhibited ‘low noise’, defined as adjacent SNPs with Δ frequency values consistently beyond the CI. Conversely, genomic regions where the sliding window mean surpassed the CI but were interspersed with numerous loci falling within the interval were interpreted as noise and subsequently excluded from further analysis. The Phenotypic Variance Explained (PVE) is calculated as the coefficient of determination based on the linear regression where the trait value (GEI BLUPs) is the dependent variable, and the genotype of the QTL is the independent variable.

## 3 Results

### 3.1 Heritability and correlation between phenotypes

**Table 1** summarizes the ranges, means, and standard errors of seed protein and the amino acids Cys, Met, Thr, and Lys concentrations (expressed as percent dry seed weight) in the two RIL populations, along with the parental means measured using NIR. In POP179, the parental lines MAA273 and MAA26 exhibited comparable mean values for all measured traits across environments. In POP180, however, MAA162 consistently showed greater mean trait values than MAA273 in most environments. All five traits displayed clear transgressive segregation in both populations relative to their respective parents.

**Table 1:** Mean, standard error (α=0.05), range, and parental means for soybean seed protein, Cys, Met, Thr, and Lys concentrations (% dry seed weight) in two RIL populations, POP179 and POP180, in four environments: Chatham 2020, Palmyra 2020, Ridgetown 2020, and Ridgetown 2021.

POP179	Trait	Environment	Mean (Std. error)	Range	MAA273	MAA26
	Protein	Chatham 2020	42.45 (0.088)	38.66 - 45.81	42.92	43.24
		Palmyra 2020	43.73 (0.125)	38.47 - 48.75	43.70	40.95
		Ridgetown 2020	43.06 (0.095)	38.95 - 47.72	41.98	42.89
		Ridgetown 2021	45.80 (0.112)	41.31 - 50.30	45.08	45.40
	Cysteine	Chatham 2020	0.56 (0.002)	0.47 - 0.62	0.57	0.59
		Palmyra 2020	0.58 (0.002)	0.50 - 0.67	0.57	0.54
		Ridgetown 2020	0.56 (0.001)	0.50 - 0.63	0.58	0.55
		Ridgetown 2021	0.55 (0.002)	0.47 - 0.69	0.57	0.54
	Methionine	Chatham 2020	0.54 (0.001)	0.50 - 0.58	0.55	0.56
		Palmyra 2020	0.57 (0.001)	0.51 - 0.63	0.56	0.54
		Ridgetown 2020	0.55 (0.001)	0.51 - 0.61	0.56	0.56
		Ridgetown 2021	0.55 (0.007)	0.46 - 0.61	0.57	0.55
	Threonine	Chatham 2020	1.58 (0.003)	1.48 - 1.67	1.58	1.59
		Palmyra 2020	1.61 (0.003)	1.43 - 1.77	1.64	1.52
		Ridgetown 2020	1.59 (0.003)	1.44 - 1.74	1.56	1.57
		Ridgetown 2021	1.65 (0.004)	1.50 - 1.80	1.65	1.65
	Lysine	Chatham 2020	2.67 (0.005)	2.45- 2.84	2.70	2.72
		Palmyra 2020	2.74 (0.007)	2.39 - 3.02	2.76	2.61
		Ridgetown 2020	2.71 (0.005)	2.47 - 2.98	2.67	2.70
		Ridgetown 2021	2.84 (0.007)	2.56 - 3.11	2.85	2.84

POP180	Trait	Environment	Mean (Std. error)	Range	MAA273	MAA162
	Protein	Chatham 2020	43.46 (0.093)	37.11 - 48.57	37.30	40.46
		Palmyra 2020	45.23 (0.136)	38.32 - 52.56	39.15	41.76
		Ridgetown 2020	44.87 (0.081)	38.02 - 51.25	38.04	41.31
		Ridgetown 2021	47.98 (0.118)	40.95 - 54.44	41.49	45.45
	Cysteine	Chatham 2020	0.59 (0.002)	0.49 - 0.65	0.51	0.54
		Palmyra 2020	0.62 (0.003)	0.54 - 0.86	0.56	0.56
		Ridgetown 2020	0.59 (0.002)	0.48 - 0.69	0.50	0.60
		Ridgetown 2021	0.61 (0.001)	0.53 - 0.70	0.54	0.59
	Methionine	Chatham 2020	0.56 (0.001)	0.49 - 0.61	0.50	0.54
		Palmyra 2020	0.59 (0.002)	0.53 - 0.67	0.54	0.57
		Ridgetown 2020	0.57 (0.002)	0.49 - 0.65	0.50	0.53
		Ridgetown 2021	0.58 (0.001)	0.52 - 0.65	0.52	0.58
	Threonine	Chatham 2020	1.60 (0.003)	1.41 - 1.76	1.41	1.50
		Palmyra 2020	1.68 (0.004)	1.50 - 1.91	1.50	1.60
		Ridgetown 2020	1.64 (0.004)	1.44 - 1.84	1.45	1.53
		Ridgetown 2021	1.71 (0.001)	1.50 - 1.90	1.51	1.66
	Lysine	Chatham 2020	2.72 (0.005)	2.38 - 3.04	2.39	2.53
		Palmyra 2020	2.85 (0.007)	2.51 - 3.23	2.53	2.68
		Ridgetown 2020	2.81 (0.008)	2.44 - 3.18	2.45	2.59
		Ridgetown 2021	2.96 (0.006)	2.58 - 3.30	2.61	2.85

**Figure 3:**
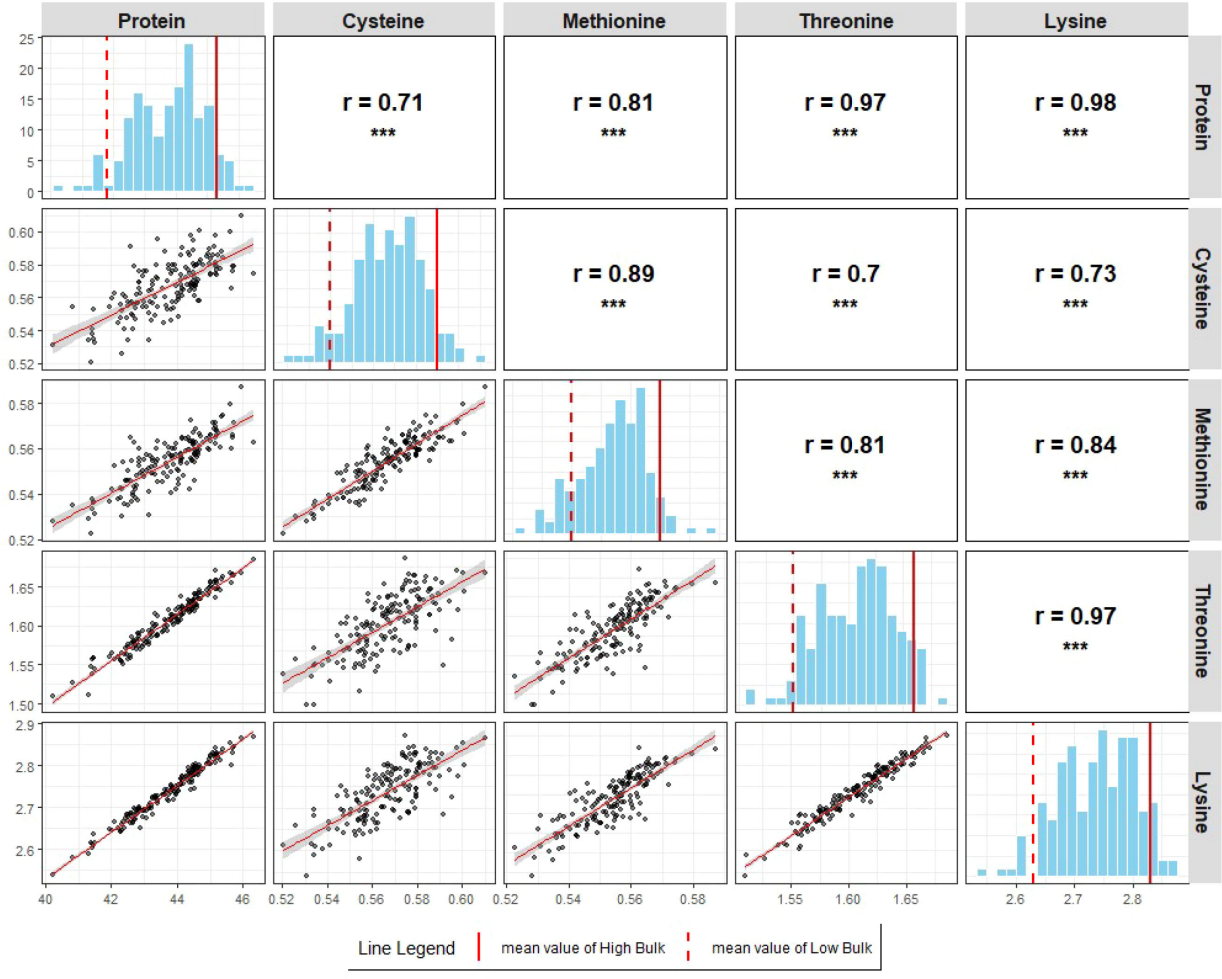
Scatterplots, histograms, and correlation matrices of seed protein, Cys, Met, Thr, and Lys concentrations (% dry seed weight) in POP179 based on GEI BLUPs. Scatterplots were drawn for all pairwise relationships with best fit line. Pearson’s correlation coefficients are calculated for all pairwise relationships (*p < 0.05, **p < 0.01, **p < 0.001). Mean values of Low and High Bulks are indicated by dashed and solid lines, respectively.

**Figure 4:**
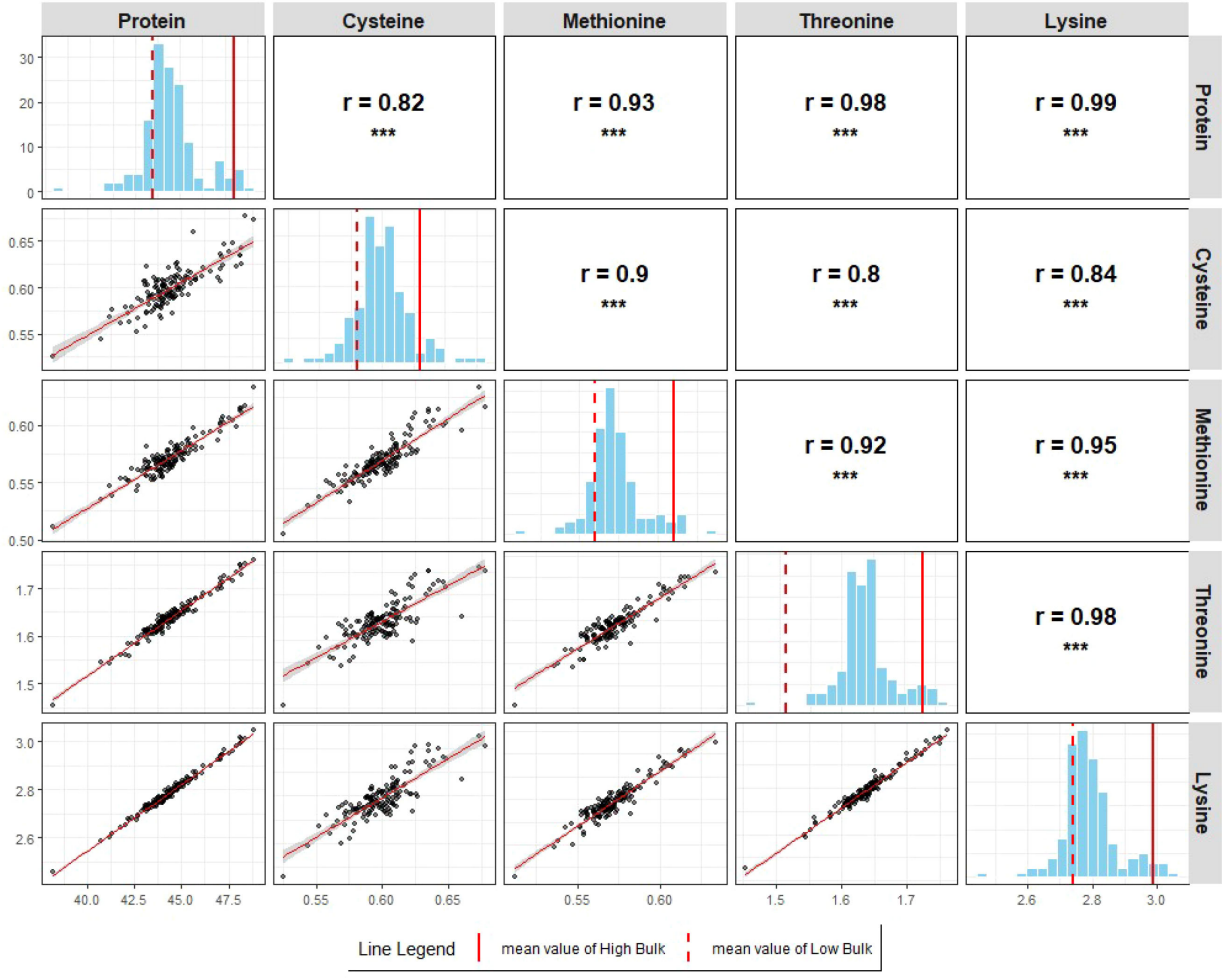
Scatterplots, histograms, and correlation matrices of seed protein, Cys, Met, Thr, and Lys concentrations (% dry seed weight) in POP180 based on GEI BLUPs. Scatterplots were drawn for all pairwise relationships with best fit line. Pearson’s correlation coefficients are calculated for all pairwise relationships (*p < 0.05, **p < 0.01, ***p < 0.001). Mean values of Low and High Bulks are indicated by dashed and solid lines, respectively.

Broad-sense heritability estimates were consistently high across all five seed component traits, with values of 0.90 for protein, 0.81 for Cys, 0.84 for Met, 0.88 for Thr, and 0.90 for Lys. Significant positive correlations (p<0.001 for all pairwise correlations; **Figures 3**, **4**) were observed among all five seed traits, measured as percent dry seed weight, in both populations. These results indicate that genotypes with higher seed protein concentrations also tend to have higher concentrations of the amino acids Cys, Met, Thr, and Lys.

### 3.2 QTL detection

In total, 62 genomic regions across 11 of the 20 soybean chromosomes were found to be associated with seed protein quantity and quality traits in the two RIL populations (**Table 2**). These regions corresponded to segments with unequal parental allele contributions, observed as peaks or troughs in the Δ SNP allele frequency plots (**Supplementary Figures 1**, **2**), and were retained only showing low noise, defined by minimal interspersion of loci with Δ allele frequencies falling within the confidence interval. Genomic regions identified in both populations are likely to be stable across genetic backgrounds and are therefore considered putative QTL (**Figures 1**, **2**; **Tables 3**, **4**).

**Table 2:** Candidate genes for soybean seed protein, cysteine, methionine, threonine, and lysine within the putative QTL interval.

Chr	Gene	Start (bp)	Stop (bp)	Functional annotation
Protein				
Gm01	*Glyma.01G091600*	27742157	27745328	Glutamine synthase
	*Glyma.01G093500*	28330727	28332206	Peptide transporter 3
	*Glyma.01G097000*	31566269	31567578	Abscisic acid receptor
Gm02	*Glyma.02G098000*	8923183	8924637	Aspartyl protease
	*Glyma.02G100800*	9493864	9495581	Aspartyl protease
Gm15	*Glyma.15G181100*	17510009	17511766	Ornithine aminotransferase
	*Glyma.15G184500*	18466448	18468257	ABC transporter G family
	*Glyma.15G186400*	19155845	19160634	Aspartic proteinase A1
	*Glyma.15G186600*	19225772	19227687	Glutamine synthetase GS1;4
	*Glyma.15G188100*	19638124	19640702	RING E3 ligase
Cysteine				
Gm15	*Glyma.15G177800*	16913568	16917215	Cysteine protease
Gm15	*Glyma.15G181100*	17510009	17511766	Ornithine aminotransferase
Methionine				
Gm02	*Glyma.02G096600*	8678024	8680049	ACT-domain amino acid regulator
Gm02	*Glyma.02G099300*	9239970	9242500	Methionine aminopeptidase 1B
Gm15	*Glyma.15G181100*	17510009	17511766	Ornithine aminotransferase
Gm15	*Glyma.15G190500*	20105420	20111720	S-adenosyl transferase
Threonine				
Gm02	*Glyma.02G098200*	8959322	8968035	Amino acid transporter
	*Glyma.02G103100*	9784634	9786935	bHLH protein with ACT domain
Lysine				
Gm02	*Glyma.02G096600*	8683532	8686231	ACT domain repeat 4
	*Glyma.02G103100*	9784634	9786935	bHLH DNA-binding protein
Gm15	*Glyma.15G191600*	20742385	20751180	Lysine-specific demethylase

Gene annotation is based on the glyma.Wm82.gnm2 assembly extracted from GlycineMine.

**Table 3:** Genomic regions associated with soybean seed protein, Cys, Met, Thr, and Lys concentrations in POP180, identified by modified QTL-seq evaluated in four environments in southern Ontario in 2020 and 2021, with their mean Δ SNP allele frequencies, confidence interval, start and end positions in basepairs.

POP180	Trait	Chr.	Mean Δ SNP allele frequency^z^	CI^y^	Start position (bp)^x^	End position (bp)^w^	Length (bp)^v^	PVE (%)^u^
	Protein	**1**	**0.14**	**0.089**	**27,089,684**	**33,126,789**	**6,037,105**	**0.12**
		**2**	**0.23**	**0.092**	** 8,288,256**	**12,920,828**	**4,632,572**	**0.11**
		6	0.20	0.062	11,748,087	14,931,242	3,183,155	1.32
		8	0.23	0.154	36,452,531	37,242,715	790,184	0.07
		9	0.05	0.026	29,806,912	34,057,105	4,250,193	0.05
		14	0.22	0.062	44,885,012	46,436,316	1,551,304	5.09
		**15**	**0.24**	**0.147**	**16,911,527**	**19,052,749**	**2,141,222**	**2.26**
		17	0.16	0.092	14,825,573	16,135,081	1,309,508	0.9
	Cysteine	14	0.22	0.021	45,182,777	45,741,873	559,096	1.97
		**15**	**0.16**	**0.068**	**16,770,964**	**19,054,691**	**2,283,727**	**2.79**
	Methionine	**2**	**0.18**	**0.067**	** 7,751,583**	**11,567,673**	**3,816,090**	**1.23**
		14	0.14	0.042	44,885,012	46,436,658	1,551,646	2.25
		**15**	**0.19**	**0.082**	**18,245,930**	**19,054,691**	**808,761**	**0.01**
	Threonine	**2**	**0.20**	**0.090**	** 8,988,050**	**12,920,828**	**3,932,778**	**0.01**
		6	0.19	0.062	12,369,739	14,931,242	2,561,503	1.8
		9	0.19	0.018	33,571,448	34,018,305	446,857	2.77
		14	0.15	0.072	44,885,012	46,436,176	1,551,164	4.61
		**15**	**0.22**	**0.136**	**17,083,965**	**19,850,837**	**2,766,872**	**2.1**
		17	0.15	0.090	14,611,480	17,067,351	2,455,871	0.55
		19	0.18	0.086	9,839,728	10,572,641	732,913	0.68
	Lysine	**2**	**0.20**	**0.086**	** 8,988,050**	**12,920,828**	**3,932,778**	**0.04**
		6	0.20	0.065	11,748,087	14,931,242	3,183,155	0.86
		9	0.11	0.015	30,234,502	36,180,587	5,946,085	0.07
		14	0.21	0.063	44,885,012	46,436,316	1,551,304	5.05
		**15**	**0.25**	**0.141**	**16,911,527**	**19,019,592**	**2,108,065**	**2.3**

^z^Calculated as the absolute mean Δ SNP allele frequency of the genomic region defined by the start and end positions.

^y^At 99.9% confidence level with degrees of freedom >1,000.

^x^Base positions of the first SNP locus with Δ SNP allele frequency beyond the confidence interval.

^w^Base positions of the last SNP locus with Δ SNP allele frequency beyond the confidence interval.

^v^Length of the genomic region in basepairs by subtracting the start position from the end position.

^u^Phenotypic variance explained by the QTL.

Bolded entries were detected in POP179 and validated in POP180 and are considered as putative QTL.

**Table 4:** Genomic regions associated with soybean seed protein, Cys, Met, Thr, and Lys concentrations in POP180, identified by modified QTL-seq evaluated in four environments in southern Ontario in 2020 and 2021, with their mean Δ SNP allele frequencies, confidence interval, start and end positions in basepairs.

POP180	Trait	Chr.	Mean Δ SNP allele frequency^z^	CI^y^	Start position (bp)^x^	End position (bp)^w^	Length (bp)^v^	PVE (%)^u^
	Protein	**1**	**0.14**	**0.089**	**27,089,684**	**33,126,789**	**6,037,105**	**0.12**
		**2**	**0.23**	**0.092**	** 8,288,256**	**12,920,828**	**4,632,572**	**0.11**
		6	0.20	0.062	11,748,087	14,931,242	3,183,155	1.32
		8	0.23	0.154	36,452,531	37,242,715	790,184	0.07
		9	0.05	0.026	29,806,912	34,057,105	4,250,193	0.05
		14	0.22	0.062	44,885,012	46,436,316	1,551,304	5.09
		**15**	**0.24**	**0.147**	**16,911,527**	**19,052,749**	**2,141,222**	**2.26**
		17	0.16	0.092	14,825,573	16,135,081	1,309,508	0.9
	Cysteine	14	0.22	0.021	45,182,777	45,741,873	559,096	1.97
		**15**	**0.16**	**0.068**	**16,770,964**	**19,054,691**	**2,283,727**	**2.79**
	Methionine	**2**	**0.18**	**0.067**	** 7,751,583**	**11,567,673**	**3,816,090**	**1.23**
		14	0.14	0.042	44,885,012	46,436,658	1,551,646	2.25
		**15**	**0.19**	**0.082**	**18,245,930**	**19,054,691**	**808,761**	**0.01**
	Threonine	**2**	**0.20**	**0.090**	** 8,988,050**	**12,920,828**	**3,932,778**	**0.01**
		6	0.19	0.062	12,369,739	14,931,242	2,561,503	1.8
		9	0.19	0.018	33,571,448	34,018,305	446,857	2.77
		14	0.15	0.072	44,885,012	46,436,176	1,551,164	4.61
		**15**	**0.22**	**0.136**	**17,083,965**	**19,850,837**	**2,766,872**	**2.1**
		17	0.15	0.090	14,611,480	17,067,351	2,455,871	0.55
		19	0.18	0.086	9,839,728	10,572,641	732,913	0.68
	Lysine	**2**	**0.20**	**0.086**	** 8,988,050**	**12,920,828**	**3,932,778**	**0.04**
		6	0.20	0.065	11,748,087	14,931,242	3,183,155	0.86
		9	0.11	0.015	30,234,502	36,180,587	5,946,085	0.07
		14	0.21	0.063	44,885,012	46,436,316	1,551,304	5.05
		**15**	**0.25**	**0.141**	**16,911,527**	**19,019,592**	**2,108,065**	**2.3**

^z^Calculated as the absolute mean Δ SNP allele frequency of the genomic region defined by the start and end positions.

^y^At 99.9% confidence level with degrees of freedom >1,000.

^x^Base positions of the first SNP locus with Δ SNP allele frequency beyond the confidence interval.

^w^Base positions of the last SNP locus with Δ SNP allele frequency beyond the confidence interval.

^v^Length of the genomic region in basepairs by subtracting the start position from the end position.

^u^Phenotypic variance explained by the QTL.

Bolded entries were detected in POP179 and validated in POP180 and are considered as putative QTL.

Three Putative QTL associated with seed protein concentration were consistently detected in both populations on chromosome 1 (27.1-32.3 Mbp), chromosome 2 (8.6-10.8 Mbp), and chromosome 15 (16.7-20.8 Mbp) (**Figures 1**, **2**; **Tables 3**, **4**). Chromosome 15 also contained a putative QTL for seed Cys concentration (16.8-19.1 Mbp). Additionally, putative QTL for Met, Thr, and Lys were identified in overlapping genomic interval on chromosome 2 (8.6-10.8 Mbp) and chromosome 15 (16.8-20.8 Mbp) in both populations (**Tables 3**, **4**).

In POP179, genomic regions associated with all five seed composition traits were detected on chromosomes 1, 2, 5, 10, 12, 15, and 20 (**Table 3**). An additional region on chromosome 1 (38.7-41.3 Mbp) was associated with both Cys and Met. Chromosome 16 harboured a region associated exclusively with Cys (7.6-10.7 Mbp), with no associations observed for the other traits.

In POP180, genomic regions associated with all five traits were found on chromosomes 14 and 15 (44.9-46.4 Mbp and 16.9-19.1 Mbp, respectively; **Table 4**); notably, the region on chromosome 14 was not detected in POP179. Additional genomic regions associated with protein, Thr, and Lys were detected on chromosomes 6 (11.7-14.9 Mbp) and 9 (29.8-34.1 Mbp) (**Table 4**). These regions were not associated with the sulfur-containing amino acids Cys and Met.

## 4 Discussion

The heritability estimate for protein was comparable to previously reported values (0.93 in Warrington et al., 2015; 0.92 and 079 in two populations reported by Wang et al., 2015). Similarly, the heritability estimates observed for Cys and Met were close to those reported by Wang et al., (0.91 and 0.87 for two populations, respectively). Collectively, these estimates indicate that variations in all five seed traits are under strong genetic control and that substantial potential exists for effective selection.

This study identified putative QTL associated with soybean seed protein and the amino acids Cys, Met, Thr, and Lys concentrations in both POP179 and POP180 (**Tables 3**, **4**, respectively). **Supplementary Table 5** listed putative QTL that overlapped with previously reported loci collected in Soybase. A notable finding was a protein QTL on chromosome 1 that did not co-localize with any previously reported loci in curated QTL databases, SoyBase (**Table 3**; Grant et al., 2010), or in the published literature, suggesting that this locus may represent a novel genetic region influencing seed protein concentration. In POP179, this QTL was also associated with all four amino acids; however, it was not validated in POP180 (**Table 3**). The candidate genes in this interval are listed in **Table 2** and include a glutamine synthetase (*Glyma.01G091600*), peptide transporter 3 (*Glyma.01G093500*), and an abscisic acid receptor (*Glyma.01G097000*). Glutamine synthetase is an important enzyme for nitrogen assimilation, responsible for the initial uptake of ammonium and is also associated with protein accumulation in seeds (Bernard and Habash, 2009). Peptide transporters are known to be critical for the long-distance transport of nitrogen-containing molecules (Tegeder, 2014). The co-localization of glutamine synthetase and peptide transporter 3 within the same interval on chromosome 1 suggests its involvement in both the synthesis and subsequent translocation of nitrogenous compounds from source tissues to the developing seed pods. The abscisic acid receptor is moderately expressed in green pods (Libault et al., 2010). Abscisic acid is a phytohormone involved in diverse developmental and stress tolerance processes. The concentration of abscisic acid in soybean seeds is known to correlate with seed growth rate (Schussler et al., 1984).

A QTL on chromosome 2 (8.6-10.8 Mbp; **Table 3**) was associated with protein, Met, Thr, and Lys. This region overlaps with two previously reported protein QTL: one by Kabelka et al. (2004; 7.1-9.5 Mbp) and another by Mao et al. (2013; 9.3-13.3 Mbp). However, there were no prior records in Soybase (Grant et al., 2010) linking this genomic region to Met, Thr, or Lys. The co-localization of these traits suggests that this region may contribute to the aspartate-derived amino acid biosynthesis pathway, which gives rise to Met, Thr, and Lys, as well as broader protein synthesis processes. This is supported by the presence of aspartyl proteases (*Glyma.02G098000*, *Glyma.02G100800*; **Table 2**) and an amino acid transporter (*Glyma.02G098200*; **Table 2**). Three candidate genes containing ACT domain are also found in this interval (*Glyma.02G096600*, *Glyma.02G096600, Glyma.02G103100*; **Table 2**). The ACT domain is regulated by amino acid concentrations and is involved in amino acid metabolism and solute transport (Grant, 2006).

A QTL located on chromosome 15 (16.7-20.8 Mbp; **Table 3**) was associated with all five seed composition traits. This region overlaps with protein QTL identified by Jun et al. (2008; 11–38 Mbp), sulfur-containing amino acids reported by Wang et al. (2015; 9.4-39.7 Mbp), and Lys QTL described by Panthee et al. (2006; 17-30.2 Mbp). A major Cys and Met QTL *qCM-15* reported by Souza et al. (2023a) is adjacent (22.17-47.17Mb). No previous studies have reported an association between this genomic region and Thr concentration. This locus includes candidate genes with numerous functions (**Table 2**). The majority are involved in amino acid synthesis and interconversion (*Glyma.15G181100*, *Glyma.15G186400, Glyma.15G181100, Glyma.15G190500, Glyma.15G191600*). The cysteine protease (*Glyma.15G177800*) and aspartic proteinase A1 (*Glyma.15G186400*) are associated with protein catabolism. The ABC transporter G (*Glyma.15G184500*) is highly expressed in green pods (Libault et al., 2010), suggesting a role in nutrient loading and seed development. Collectively, this locus may be involved in amino acid biosynthesis, protein catabolism, and nutrient loading in seeds.

In POP179, genomic regions on chromosomes 1, 2, 5, 10, 15, and 20 were associated with all five seed traits - protein, Cys, Met, Thr, and Lys (**Table 3**), indicating a shared genetic basis among these composition traits. The locus on chromosome 10 was adjacent to the major Cys and Met QTL *qCM-10* reported by Souza et al. (2023a; 3.03–3.98Mb). The region on chromosome 20 (11.8-31.3 Mbp; **Table 3**) was particularly notable due to its extended span of nearly 20 Mbp. Wang et al. (2021) previously identified a broader overlapping region on chromosome 20 (0.01-37.89 Mbp) using traditional QTL-seq and further resolved this into two adjacent QTL using linkage mapping. Chromosome 20 has been repeatedly shown to harbour QTL for seed protein and multiple amino acids, with numerous studies reporting co-localization of these traits within this genomic interval (Lu et al., 2013; Mao et al., 2013; Panthee et al., 2006; Pandurangan et al., 2012; Wang et al., 2015; Warrington et al., 2015; Malle et al., 2020; Wang et al., 2021; Hong et al., 2022a). Similarly, in POP180, genomic regions on chromosomes 14 and 15 were associated with all five traits (**Table 4**), further supporting the presence of pleiotropic loci or tightly linked genes governing seed composition.

Most genomic regions identified in this study exhibited relatively low PVE values, as listed in **Tables 3**, **4**. This demonstrated the complex and polygenic architecture of soybean seed composition where individual amino acid concentrations are regulated by numerous loci. Despite the overall low PVE values, these findings provide critical information for developing targeted breeding strategies to enhance seed quality. Genomic regions with comparatively higher effects, such as the QTL on chromosome 5 (5.17% PVE), represent a potential candidate for the development of markers to facilitate MAS for seed protein quality and quantity improvements. Conversely, for the broader collection of minor effect loci, a more effective strategy may involve integrating these validated QTL into whole-genome prediction models. Recent research utilizing diverse soybean panels has shown that while genomic prediction remains superior for seed protein and oil content, the inclusion of specific marker-trait associations as weighted features can address the limitations of MAS for traits governed by complex genetic architectures (van der Laan et al., 2025). Hence, utilizing specific genomic regions with low PVE as fixed effects or weighted features within genomic selection programs could still enhance the accuracy of predicting seed quality traits across diverse populations, ultimately supporting the development of cultivars with superior nutritional profiles.

While the parental lines MAA26, MAA273, and MAA162 exhibited relatively similar mean concentrations for Cys, Met, Thr, and Lys (**Supplementary Table 1**), distinct phenotypic variations were found in both POP179 and POP180. Offspring individuals with phenotypic values exceeding the parental range is indictive of transgressive segregation. This could be the result of parents contributing separate and complementary alleles that are recombined in the offsprings. A previous study in cotton showed that crosses between parents that are both susceptible to root-knot nematodes produced highly resistant offsprings through the accumulation of complementary beneficial alleles (Wang et al., 2012). Furthermore, research in rice has shown that similar parental phenotypes can result from a balance of multiple minor effect QTL with opposing additive effects (Koide et al., 2019). This balance is disrupted by recombination and produces transgressive segregation in the offsprings (Koide et al., 2019). Given that the parents in this study were derived from fast neutron mutants, it is possible that they have different mutations that affect separate regulatory or metabolic processes related to seed composition. This underlying difference is only revealed after recombination, allowing for the detection of significant QTL on chromosomes 1, 2, and 15, despite the similar phenotypic values among the parents.

The genetic relationships among all five seed composition traits were supported by phenotypic correlation analyses. In this study, significant positive correlations were observed among seed protein, Cys, Met, Thr, and Lys concentrations, expressed as percent dry seed weight, in both RIL populations (**Figures 3**, **4** for POP179 and POP180, respectively). Previous studies have reported inconsistent relationships between protein and amino acid composition. For example, Wilcox and Shibles (2001) observed no correlation between protein and the sulfur-containing amino acids, whereas Panthee et al. (2006) reported a negative correlation between protein and Cys, and no correlation with Met. In contrast, subsequent studies reported positive correlations between protein and Cys, Met, Thr, and Lys (Warrington et al., 2014; Hong et al., 2022b). Pfarr et al. (2018) reported that Cys, Met, Thr, and Lys concentrations decrease as seed protein concentration increases, suggesting a trade-off between protein quantity and quality. While Singer et al. (2022) highlighted the challenge of improving methionine concentration due to its low natural abundance in soybean protein, the discovery of QTLs on chromosomes 2 and 15 that concurrently enhance protein and specific amino acid profiles may address this limitation.

The positive correlations among all five seed traits, expressed as percent dry seed weight, reflect the shared biosynthetic and compositional relationships among seed proteins and the amino acids. In soybean, protein typically comprises 30 to 40% of the dry seed weight (Banaszkiewicz, 2011), and a substantial proportion of this protein is composed of globulins, which function as amino acid reserves that are mobilized through hydrolysis during germination (Kim et al., 2011). The 7S β-conglycinin and 11S glycinin storage proteins together account for more than 70% of the globulin fraction in soybean seed ((Murphy and Resurreccion, 1984; Yaklich et al., 1999). Notably, Cys and Met represent more than 3% of amino acid residues in 11S globulins but less than 1% in 7S fraction (Sexton et al., 1998), highlighting differences in sulfur amino acids composition between the two major storage proteins. The discovery of three QTL associated with 11S glycinin by Boehm et al. (2018) offers an alternative breeding strategy for enhancing cysteine and methionine levels through the targeted selection of 11S storage protein fractions. Met, Thr, and Lys are synthesized via the aspartate-derived pathway, while Cys acts as the sulfur donor required for Met biosynthesis (Sexton et al., 2002). Hence, the observed positive correlations among protein, Cys, Met, Thr, and Lys concentrations align with their interconnected roles in storage protein composition and amino acid biosynthesis pathways.

The persistent negative correlations of protein with both yield and oil are well-documented (Cober and Voldeng, 2000; Wilcox and Shibles, 2001; Yin and Vyn, 2005; Hwang et al., 2014; de Borja Reis et al., 2020; Hong et al., 2022a). Major protein QTL on chromosome 20 co-localize with oil QTL with opposing additive effect (Hwang et al., 2014). This suggests that these traits are either controlled by a single pleiotropic gene or by separate, tightly linked genes that are difficult to separate through recombination. These findings complicate the development of new cultivars with improved protein content and profiles and may limit their economic potential. The high-protein cultivar “Danbaekkong” (PI 619083; Kim et al., 1996) contains a high protein QTL first reported by Warrington et al. (2015). By introgressing the favorable allele from this locus into the elite background ‘Benning’ (PI 595645; Boerma et al., 1997), Prenger et al. (2019) created high-protein lines that overcame the traditional yield drag. This high-protein allele originated from a *Glycine soja* line (Souza et al., 2023b). These findings highlight the potential for identifying novel sources beneficial alleles within exotic populations. Utilizing these diverse backgrounds may enable the development of cultivars with superior protein profiles while maintaining yield.

This study identified putative QTL associated with seed protein quantity and quality traits in novel genetic backgrounds derived from fast-neutron mutant lines. We implemented a modified QTL-seq framework, based on the original method described by Takagi et al. (2013), which has seen increasing use in recent years (Singh et al., 2016; Park et al., 2019; da Silva et al., 2019; Wang et al., 2021). Using this approach, we detected and validated three genomic regions harbouring putative QTL associated with one or more of the five seed composition traits across our RIL populations. Notably, a region on chromosome 1 contained a putative protein QTL that did not co-localize with loci previously reported in SoyBase (Grant et al., 2010) or in the broader literature, suggesting it may represent a novel genetic region influencing seed protein concentration.

## 5 Conclusion

In this study, we applied a modified QTL-seq approach to investigate the genetic basis of soybean seed protein quantity and quality traits in unique mutant-derived RIL populations. By utilizing unique mutant-derived parents, this study characterized loci that may contribute to seed protein quantity and quality traits in novel genetic backgrounds not previously used in conventional elite-by-elite crosses. Putative QTL associated with protein and key amino acids, Cys, Met, Thr, and Lys, were identified on chromosomes 1, 2, and 15, including loci not previously reported in public databases or the broader literature. These regions represent promising targets for breeding and may contain novel genetic contributors to seed composition.

## Data Availability

The datasets analyzed for this study can be found in the Figshare repository: https://doi.org/10.6084/m9.figshare.31395318.v1.
