# Supplementary material for: QTL-seq analysis of seed protein quantity and quality traits in two soybean recombinant inbred line populations
Source: Front Plant Sci. 2026 Apr 15;17:1771028. doi: 10.3389/fpls.2026.1771028 (PMC13125124; doi:10.3389/fpls.2026.1771028)
Supplement: Supplementary file 1 [file Table1.docx]

**Supplementary Table 1**: Mean concentrations of seed cysteine, methionine, threonine, and lysine (percent dry seed weight) of three parental lines, MAA26, MAA273, and MAA 162, as well as the minimum and maximum values of POP179 and POP180, grown in Ridgetown, 2019 (data not used).

|  | **Measured Values (% dry seed weight)** | | | | |
| --- | --- | --- | --- | --- | --- |
| **Amino Acid** | **MAA26** | **MAA273** | **MAA162** | **POP179 (min-max)** | **POP180 (min-max)** |
| **Cysteine** | 0.79 | 0.76 | 0.73 | 0.64-0.51 | 0.67-0.53 |
| **Methionine** | 0.67 | 0.67 | 0.67 | 0.63-0.52 | 0.68-0.53 |
| **Threonine** | 1.83 | 1.78 | 1.85 | 1.74-1.47 | 1.89-1.54 |
| **Lysine** | 3.24 | 3.21 | 3.27 | 3.01-2.55 | 3.28-2.61 |

**Supplementary Table 2**: Measured concentrations of seed protein, cysteine, methionine, threonine, and lysine (percent dry seed weight) from POP179 and POP180 grown in Ridgetown, 2019 (data not used).

| **Seed Component** | **Measured Values (% dry seed weight)** | | |
| --- | --- | --- | --- |
|  |  | **POP179** | **POP180** |
| **Protein** | Max | 48.86 | 52.52 |
|  | Mean | 43.88 | 45.84 |
|  | Min | 40.27 | 41.08 |
|  | **St. Dev.** | **1.72** | **2.15** |
| **Cysteine** | Max | 0.64 | 0.67 |
|  | Mean | 0.57 | 0.58 |
|  | Min | 0.51 | 0.53 |
|  | **St. Dev.** | **0.02** | **0.02** |
| **Methionine** | Max | 0.63 | 0.68 |
|  | Mean | 0.57 | 0.58 |
|  | Min | 0.52 | 0.53 |
|  | **St. Dev.** | **0.0** | **0.03** |
| **Threonine** | Max | 1.74 | 1.89 |
|  | Mean | 1.59 | 1.66 |
|  | Min | 1.47 | 1.54 |
|  | **St. Dev.** | **0.06** | **0.07** |
| **Lysine** | Max | 3.01 | 3.28 |
|  | Mean | 2.61 | 2.86 |
|  | Min | 2.55 | 2.61 |
|  | **St. Dev.** | **0.09** | **0.13** |

**Supplementary Table 3**: Individual recombinant inbred lines that were selected to form the Low and High Bulks for POP179 for the traits seed protein, cysteine, methionine, threonine, and lysine. Individuals are listed with their corresponding combined BLUPs (% dry seed weight) calculated based on multi-environment trials in Chatham 2020, Palmyra 2020, Ridgetown 2020, and Ridgetown 2021.

| **Trait** | **Low Bulk** | | **High Bulk** | |
| --- | --- | --- | --- | --- |
|  | **Genotype** | **Combined BLUPs** | **Genotype** | **Combined BLUPs** |
| **Protein** | 179-10 | 41.48 | 179-7 | 44.96 |
|  | 179-28 | 41.42 | 179-15 | 46.34 |
|  | 179-47 | 42.04 | 179-38 | 45.93 |
|  | 179-51 | 41.40 | 179-45 | 45.30 |
|  | 179-53 | 41.96 | 179-46 | 45.68 |
|  | 179-56 | 40.81 | 179-63 | 45.69 |
|  | 179-58 | 41.45 | 179-69 | 45.64 |
|  | 179-60 | 42.09 | 179-72 | 45.16 |
|  | 179-82 | 42.43 | 179-81 | 44.81 |
|  | 179-86 | 42.34 | 179-87 | 45.02 |
|  | 179-88 | 40.21 | 179-120 | 45.37 |
|  | 179-90 | 42.29 | 179-127 | 44.95 |
|  | 179-91 | 41.47 | 179-133 | 45.21 |
|  | 179-114 | 42.39 | 179-135 | 44.99 |
|  | 179-118 | 41.44 | 179-139 | 45.10 |
|  | 179-121 | 41.31 | 179-141 | 45.16 |
|  | 179-123 | 42.65 | 179-144 | 45.60 |
|  | 179-130 | 42.21 | 179-153 | 45.35 |
|  | 179-157 | 42.29 | 179-155 | 45.00 |
|  | 179-158 | 42.32 | 179-156 | 45.18 |
|  | **Mean** | **41.80** |  | **45.32** |
| **Cysteine** | 179-10 | 0.53 | 179-2 | 0.60 |
|  | 179-16 | 0.55 | 179-6 | 0.60 |
|  | 179-25 | 0.55 | 179-21 | 0.58 |
|  | 179-28 | 0.54 | 179-27 | 0.59 |
|  | 179-36 | 0.53 | 179-29 | 0.59 |
|  | 179-51 | 0.52 | 179-38 | 0.61 |
|  | 179-59 | 0.53 | 179-68 | 0.59 |
|  | 179-78 | 0.55 | 179-70 | 0.60 |
|  | 179-85 | 0.54 | 179-94 | 0.59 |
|  | 179-88 | 0.53 | 179-120 | 0.58 |
|  | 179-90 | 0.55 | 179-125 | 0.58 |
|  | 179-91 | 0.53 | 179-127 | 0.59 |
|  | 179-106 | 0.55 | 179-135 | 0.59 |
|  | 179-114 | 0.54 | 179-138 | 0.59 |
|  | 179-118 | 0.54 | 179-139 | 0.59 |
|  | 179-121 | 0.53 | 179-141 | 0.58 |
|  | 179-123 | 0.55 | 179-142 | 0.59 |
|  | 179-128 | 0.54 | 179-144 | 0.60 |
|  | 179-130 | 0.54 | 179-153 | 0.59 |
|  | 179-158 | 0.55 | 179-156 | 0.60 |
|  | **Mean** | **0.54** |  | **0.59** |
| **Methionine** | 179-10 | 0.53 | 179-2 | 0.57 |
|  | 179-16 | 0.54 | 179-3 | 0.57 |
|  | 179-28 | 0.54 | 179-27 | 0.57 |
|  | 179-36 | 0.53 | 179-38 | 0.59 |
|  | 179-51 | 0.52 | 179-72 | 0.57 |
|  | 179-53 | 0.54 | 179-98 | 0.57 |
|  | 179-56 | 0.53 | 179-120 | 0.57 |
|  | 179-58 | 0.54 | 179-132 | 0.57 |
|  | 179-59 | 0.53 | 179-133 | 0.57 |
|  | 179-88 | 0.53 | 179-135 | 0.56 |
|  | 179-90 | 0.54 | 179-137 | 0.57 |
|  | 179-91 | 0.54 | 179-139 | 0.57 |
|  | 179-102 | 0.54 | 179-140 | 0.56 |
|  | 179-106 | 0.54 | 179-141 | 0.56 |
|  | 179-114 | 0.54 | 179-142 | 0.57 |
|  | 179-118 | 0.53 | 179-144 | 0.58 |
|  | 179-121 | 0.53 | 179-151 | 0.57 |
|  | 179-128 | 0.54 | 179-153 | 0.57 |
|  | 179-130 | 0.54 | 179-155 | 0.57 |
|  | 179-158 | 0.54 | 179-156 | 0.57 |
|  | **Mean** | **0.54** |  | **0.57** |
| **Threonine** | 179-10 | 1.54 | 179-3 | 1.66 |
|  | 179-28 | 1.54 | 179-7 | 1.65 |
|  | 179-47 | 1.56 | 179-15 | 1.69 |
|  | 179-51 | 1.55 | 179-35 | 1.66 |
|  | 179-56 | 1.53 | 179-38 | 1.67 |
|  | 179-58 | 1.56 | 179-45 | 1.65 |
|  | 179-59 | 1.56 | 179-46 | 1.66 |
|  | 179-60 | 1.56 | 179-63 | 1.67 |
|  | 179-82 | 1.57 | 179-87 | 1.65 |
|  | 179-86 | 1.55 | 179-98 | 1.66 |
|  | 179-88 | 1.51 | 179-120 | 1.66 |
|  | 179-90 | 1.56 | 179-131 | 1.64 |
|  | 179-91 | 1.56 | 179-133 | 1.65 |
|  | 179-112 | 1.56 | 179-139 | 1.65 |
|  | 179-118 | 1.56 | 179-141 | 1.65 |
|  | 179-121 | 1.51 | 179-144 | 1.67 |
|  | 179-143 | 1.57 | 179-151 | 1.66 |
|  | 179-148 | 1.57 | 179-153 | 1.66 |
|  | 179-157 | 1.56 | 179-155 | 1.65 |
|  | 179-158 | 1.55 | 179-156 | 1.67 |
|  | **Mean** | **1.55** |  | **1.66** |
| **Lysine** | 179-10 | 2.62 | 179-3 | 2.82 |
|  | 179-28 | 2.61 | 179-15 | 2.87 |
|  | 179-47 | 2.64 | 179-35 | 2.82 |
|  | 179-51 | 2.61 | 179-38 | 2.86 |
|  | 179-56 | 2.58 | 179-46 | 2.83 |
|  | 179-58 | 2.64 | 179-63 | 2.84 |
|  | 179-60 | 2.67 | 179-72 | 2.81 |
|  | 179-86 | 2.66 | 179-87 | 2.82 |
|  | 179-88 | 2.54 | 179-98 | 2.83 |
|  | 179-90 | 2.64 | 179-120 | 2.84 |
|  | 179-91 | 2.61 | 179-132 | 2.80 |
|  | 179-114 | 2.65 | 179-133 | 2.83 |
|  | 179-118 | 2.60 | 179-135 | 2.82 |
|  | 179-121 | 2.59 | 179-139 | 2.83 |
|  | 179-123 | 2.66 | 179-141 | 2.82 |
|  | 179-128 | 2.67 | 179-144 | 2.85 |
|  | 179-130 | 2.65 | 179-151 | 2.83 |
|  | 179-148 | 2.65 | 179-153 | 2.83 |
|  | 179-157 | 2.64 | 179-155 | 2.82 |
|  | 179-158 | 2.65 | 179-156 | 2.84 |
|  | **Mean** | **2.63** |  | **2.83** |

**Supplementary Table 4**: Individual recombinant inbred lines that were selected to form the Low and High Bulks for POP180 for the traits seed protein, cysteine, methionine, threonine, and lysine. Individuals are listed with their corresponding combined BLUPs (% dry seed weight) calculated based on multi-environment trials in Chatham 2020, Palmyra 2020, Ridgetown 2020, and Ridgetown 2021.

| **Trait** | **Low Bulk** | | **High Bulk** | |
| --- | --- | --- | --- | --- |
| **Protein** | **Genotype** | **Combined BLUPs** | **Genotype** | **Combined BLUPs** |
|  | 180-6 | 43.45 | 180-9 | 48.75 |
|  | 180-8 | 41.61 | 180-47 | 46.90 |
|  | 180-10 | 43.17 | 180-49 | 48.61 |
|  | 180-12 | 43.85 | 180-50 | 47.22 |
|  | 180-19 | 43.88 | 180-63 | 47.88 |
|  | 180-20 | 44.12 | 180-73 | 47.07 |
|  | 180-26 | 43.24 | 180-101 | 48.85 |
|  | 180-29 | 43.82 | 180-107 | 47.60 |
|  | 180-30 | 43.40 | 180-111 | 48.58 |
|  | 180-31 | 43.21 | 180-112 | 47.95 |
|  | 180-43 | 43.94 | 180-115 | 47.69 |
|  | 180-51 | 44.18 | 180-116 | 48.59 |
|  | 180-53 | 41.87 | 180-118 | 48.60 |
|  | 180-70 | 42.33 | 180-119 | 48.56 |
|  | 180-79 | 44.24 | 180-120 | 48.32 |
|  | 180-95 | 44.10 | 180-124 | 47.94 |
|  | 180-100 | 44.24 | 180-125 | 47.46 |
|  | 180-113 | 44.09 | 180-128 | 48.39 |
|  | 180-133 | 43.34 | 180-136 | 47.16 |
|  | 180-134 | 43.83 | 180-138 | 48.56 |
|  | **Mean** | **43.50** |  | **48.03** |
| **Cysteine** | 180-6 | 0.58 | 180-9 | 0.66 |
|  | 180-8 | 0.55 | 180-40 | 0.65 |
|  | 180-22 | 0.57 | 180-49 | 0.64 |
|  | 180-30 | 0.57 | 180-63 | 0.64 |
|  | 180-31 | 0.57 | 180-73 | 0.62 |
|  | 180-33 | 0.59 | 180-75 | 0.63 |
|  | 180-42 | 0.59 | 180-97 | 0.62 |
|  | 180-53 | 0.57 | 180-101 | 0.63 |
|  | 180-57 | 0.59 | 180-107 | 0.62 |
|  | 180-61 | 0.59 | 180-111 | 0.62 |
|  | 180-67 | 0.59 | 180-112 | 0.62 |
|  | 180-70 | 0.56 | 180-116 | 0.63 |
|  | 180-72 | 0.59 | 180-118 | 0.66 |
|  | 180-98 | 0.59 | 180-119 | 0.62 |
|  | 180-100 | 0.59 | 180-120 | 0.64 |
|  | 180-106 | 0.59 | 180-124 | 0.63 |
|  | 180-113 | 0.58 | 180-125 | 0.64 |
|  | 180-117 | 0.59 | 180-128 | 0.64 |
|  | 180-131 | 0.58 | 180-135 | 0.63 |
|  | 180-134 | 0.58 | 180-138 | 0.63 |
|  | **Mean** | **0.58** |  | **0.63** |
| **Methionine** | 180-6 | 0.56 | 180-9 | 0.61 |
|  | 180-7 | 0.56 | 180-47 | 0.59 |
|  | 180-8 | 0.54 | 180-49 | 0.61 |
|  | 180-10 | 0.56 | 180-50 | 0.59 |
|  | 180-13 | 0.56 | 180-63 | 0.60 |
|  | 180-16 | 0.58 | 180-73 | 0.60 |
|  | 180-30 | 0.55 | 180-101 | 0.61 |
|  | 180-31 | 0.55 | 180-107 | 0.60 |
|  | 180-42 | 0.56 | 180-111 | 0.61 |
|  | 180-43 | 0.56 | 180-112 | 0.61 |
|  | 180-53 | 0.54 | 180-115 | 0.60 |
|  | 180-56 | 0.56 | 180-116 | 0.61 |
|  | 180-57 | 0.56 | 180-118 | 0.62 |
|  | 180-70 | 0.55 | 180-119 | 0.60 |
|  | 180-100 | 0.56 | 180-120 | 0.61 |
|  | 180-113 | 0.56 | 180-124 | 0.61 |
|  | 180-117 | 0.55 | 180-125 | 0.60 |
|  | 180-131 | 0.56 | 180-128 | 0.61 |
|  | 180-133 | 0.56 | 180-136 | 0.59 |
|  | 180-134 | 0.56 | 180-138 | 0.61 |
|  | **Mean** | **0.56** |  | **0.61** |
| **Threonine** | 180-6 | 1.60 | 180-9 | 1.76 |
|  | 180-8 | 1.57 | 180-47 | 1.71 |
|  | 180-10 | 1.61 | 180-49 | 1.74 |
|  | 180-17 | 1.63 | 180-50 | 1.71 |
|  | 180-19 | 1.63 | 180-63 | 1.73 |
|  | 180-26 | 0.59 | 180-73 | 1.71 |
|  | 180-29 | 0.61 | 180-101 | 1.76 |
|  | 180-30 | 1.60 | 180-107 | 1.72 |
|  | 180-31 | 1.59 | 180-111 | 1.75 |
|  | 180-43 | 1.62 | 180-112 | 1.74 |
|  | 180-53 | 1.56 | 180-115 | 1.72 |
|  | 180-70 | 1.57 | 180-116 | 1.76 |
|  | 180-72 | 1.63 | 180-118 | 1.76 |
|  | 180-79 | 1.63 | 180-119 | 1.74 |
|  | 180-113 | 1.62 | 180-120 | 1.73 |
|  | 180-129 | 1.64 | 180-124 | 1.72 |
|  | 180-131 | 1.62 | 180-125 | 1.73 |
|  | 180-133 | 1.60 | 180-128 | 1.74 |
|  | 180-134 | 1.62 | 180-136 | 1.71 |
|  | 180-137 | 1.64 | 180-138 | 1.76 |
|  | **Mean** | **1.51** |  | **1.73** |
| **Lysine** | 180-6 | 2.72 | 180-9 | 3.03 |
|  | 180-8 | 2.63 | 180-47 | 2.93 |
|  | 180-10 | 2.73 | 180-49 | 3.01 |
|  | 180-17 | 2.78 | 180-50 | 2.93 |
|  | 180-19 | 2.77 | 180-63 | 2.98 |
|  | 180-20 | 2.77 | 180-73 | 2.94 |
|  | 180-26 | 2.73 | 180-101 | 3.04 |
|  | 180-29 | 2.76 | 180-107 | 2.95 |
|  | 180-30 | 2.72 | 180-111 | 3.02 |
|  | 180-31 | 2.72 | 180-112 | 2.99 |
|  | 180-43 | 2.76 | 180-115 | 2.97 |
|  | 180-53 | 2.65 | 180-116 | 3.02 |
|  | 180-70 | 2.67 | 180-118 | 3.04 |
|  | 180-72 | 2.77 | 180-119 | 3.01 |
|  | 180-79 | 2.79 | 180-120 | 3.00 |
|  | 180-100 | 2.77 | 180-124 | 2.98 |
|  | 180-113 | 2.76 | 180-125 | 2.97 |
|  | 180-131 | 2.77 | 180-128 | 3.00 |
|  | 180-133 | 2.73 | 180-136 | 2.93 |
|  | 180-134 | 2.75 | 180-138 | 3.04 |
|  | **Mean** | **2.74** |  | **2.99** |

**Supplementary Table 5:** Comparison of putative QTL with previously reported loci.

| **Trait** | **Chromosome** | **Start (bp)** | **End (bp)** | **QTL name** | **Reference** |
| --- | --- | --- | --- | --- | --- |
| **Protein** | 1 | 27089684 | 32378053 |  |  |
|  | 2 | 8608657 | 10791913 | Seed protein 21-4 | Kabelka et al., 2004 |
|  |  |  |  | Seed protein 36-12 | Mao et al., 2013 |
|  | 15 | 16779008 | 20806562 | Seed protein 41-2 | Jun et al., 2008 |
|  |  |  |  | Seed protein 30-3 | Tajuddin et al., 2003; Zhang et al., 2019 |
| **Cysteine** | 15 | 16779008 | 20816370 | Seed Cys 3-6 | Wang et al., 2015 |
| **Methionine** | 2 | 8608469 | 10791913 |  |  |
|  | 15 | 16779008 | 20806562 | Seed Met 3-6 | Wang et al., 2015 |
| **Threonine** | 2 | 8608469 | 10791913 |  |  |
|  | 15 | 16796174 | 20816370 |  |  |
| **Lysine** | 2 | 8608469 | 10791913 |  |  |
|  | 15 | 16779008 | 20816370 | Seed Lys 1-2 | Panthee et al., 2006 |
