# Supplementary material for: QTL-seq analysis of seed protein quantity and quality traits in two soybean recombinant inbred line populations
Source: Front Plant Sci. 2026 Apr 15;17:1771028. doi: 10.3389/fpls.2026.1771028 (PMC13125124; doi:10.3389/fpls.2026.1771028)
Supplement: Supplementary file 2 [file DataSheet1.docx]

Supplementary


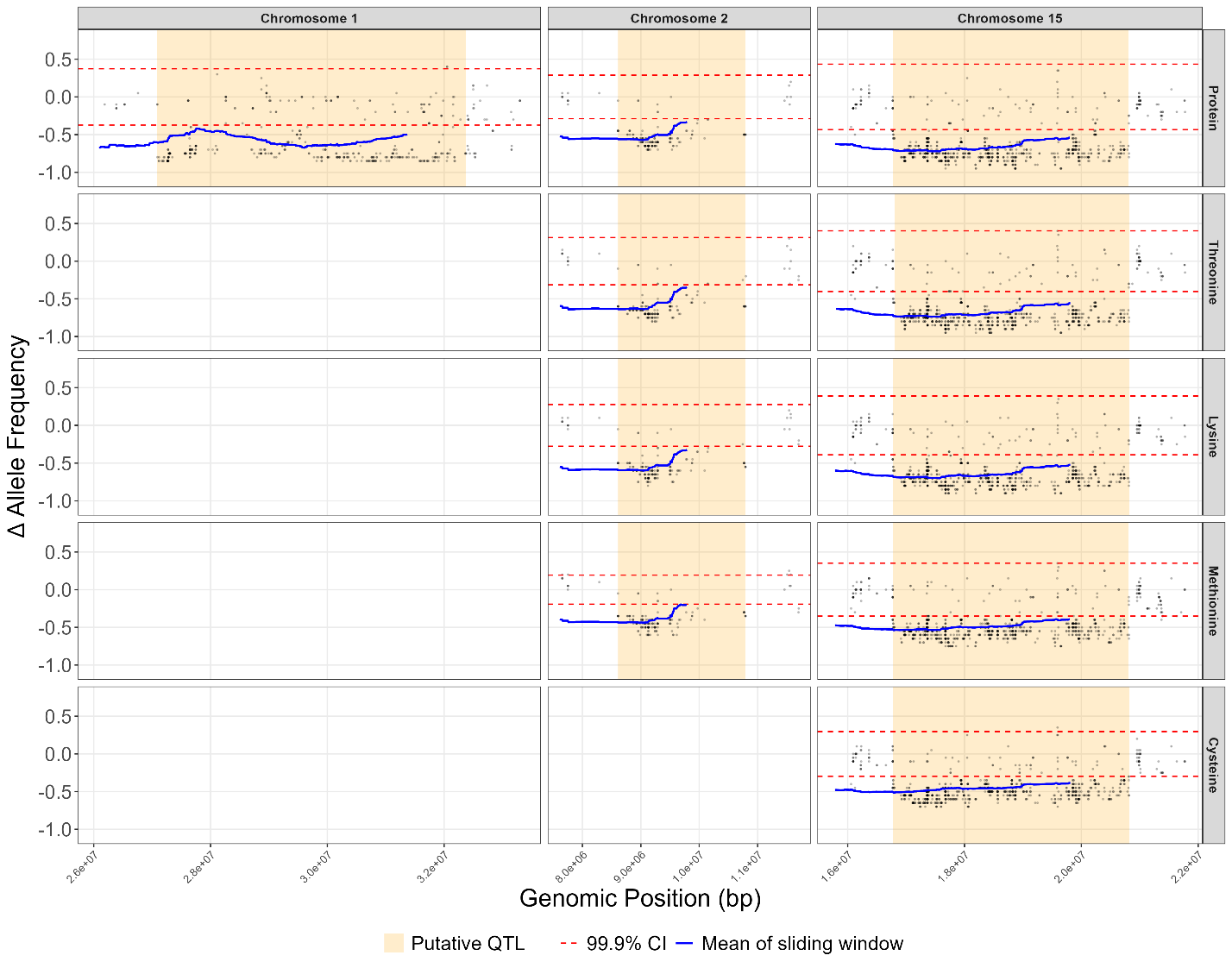


**Supplementary Figure 1**: Modified QTL-seq analysis of soybean seed composition on chromosomes 1, 2, and 15 in POP179. Panels display the Δ Single Nucleotide (SNP) allele frequency for protein, threonine, lysine, methionine, and cysteine concentrations. The genomic regions included ±1 Mb of flanking sequence surrounding the putative QTL intervals. The x-axis depicts the chromosomal position of the allele in mega-basepairs (Mb). The y-axis represents ΔSNP allele frequencies of each locus, obtained by subtracting the SNP allele frequency in the low bulk from that of the high bulk. The blue line represents the sliding-window mean of the ΔSNP allele frequency. The individual grey points represent the ΔSNP allele frequencies of specific SNPs. The orange shaded regions indicate the putative QTL intervals. Red dashed lines represent the 99.9% confidence interval (CI). Vertical alignment of panels across traits highlights significant co-localization of QTL across traits.

**
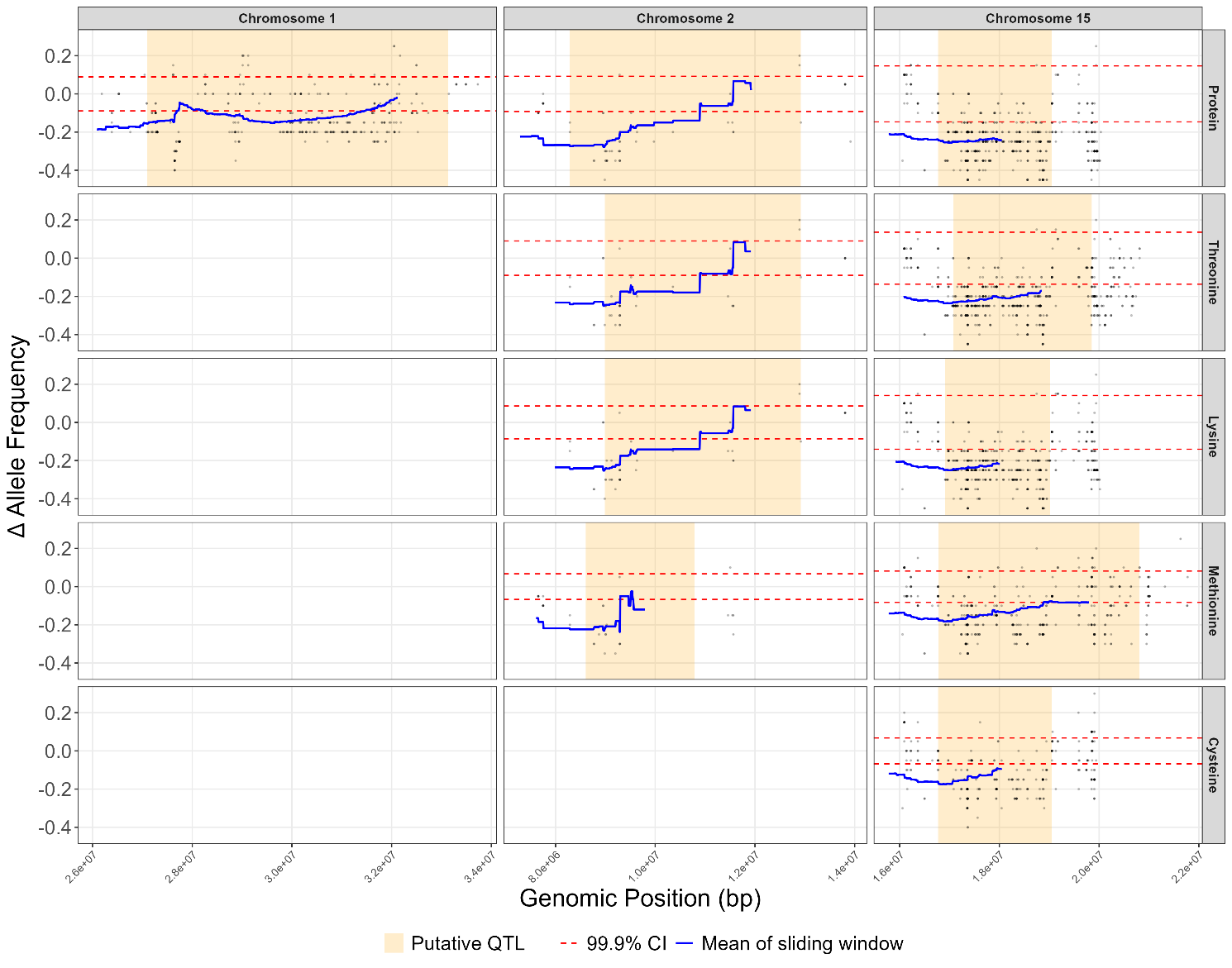
**

**Supplementary Figure 2** Modified QTL-seq analysis of soybean seed composition on chromosomes 1, 2, and 15 in POP180. Panels display the Δ Single Nucleotide (SNP) allele frequency for protein, threonine, lysine, methionine, and cysteine concentrations. The genomic regions included ±1 Mb of flanking sequence surrounding the putative QTL intervals. The x-axis depicts the chromosomal position of the allele in mega-basepairs (Mb). The y-axis represents ΔSNP allele frequencies of each locus, obtained by subtracting the SNP allele frequency in the low bulk from that of the high bulk. The blue line represents the sliding-window mean of the ΔSNP allele frequency. The individual grey points represent the ΔSNP allele frequencies of specific SNPs. The orange shaded regions indicate the putative QTL intervals. Red dashed lines represent the 99.9% confidence interval (CI). Vertical alignment of panels across traits highlights significant co-localization of QTL across traits.
